# Deposition of Diamond Coatings on Ultrathin Microdrills for PCB Board Drilling

**DOI:** 10.3390/ma17225593

**Published:** 2024-11-15

**Authors:** Shuangqing Zhou, Stephan Handschuh-Wang, Tao Wang

**Affiliations:** 1 Advanced Energy Storage Technology Center, Shenzhen Institutes of Advanced Technology, Chinese Academy of Sciences, Shenzhen 518055, China; 2 College of New Materials and New Energies, Shenzhen Technology University, Shenzhen 518118, China

**Keywords:** diamond coating, microdrills, PCB, nanocrystalline diamond, wear resistance

## Abstract

The drilling of State-of-the-Art printed circuit boards (PCBs) often leads to shortened tool lifetime and low drilling accuracy due to improved strength of the PCB composites with nanofillers and higher thickness-to-hole diameter ratio. Diamond coatings have been employed to improve the tool lifetime and drilling accuracy, but the coated microdrills are brittle and suffer from coating delamination. To date, it is still difficult to deposit diamonds on ultrathin microdrills with diameters lower than 0.2 mm. To avoid tool failure, the pretreatment was optimized to afford sufficient fracture strength and enough removal of cobalt. Further, the adhesion of the diamond coating was improved by employing an interlayer comprising SiC/microcrystalline diamond, which mitigates stress accumulation at the interface. By these means, microdrills with diameters of 0.8 and 0.125 mm were coated with adherent diamonds. In this context, the composite coating with the diamond/SiC interlayer and a nanodiamond top layer featured enhanced adhesion compared to single nano- or microdiamond coatings on the WC-Co microdrills. The composite diamond-coated WC-Co microdrills featured improved wear resistance, resistance to delamination of the diamond coating, and improved performance for drilling PCBs compared to micro- and nanodiamond-coated microdrills without interlayer. In addition, a higher hole quality was achieved when the diamond-coated microdrills were used. These results signify that the composite/nanodiamond coating features the highest bonding strength and best drilling performance.

## 1. Introduction

Printed circuit boards (PCBs) are ubiquitous in electronic devices ranging from consumer electronics, such as computers, gaming consoles, smartphones, televisions, amplifiers, and diverse control electronics in cars, via industrial applications, i.e., control devices for industrial processes, medical devices, such as hearing aids, heart rate monitors, magnetic resonance imaging (MRI) and computed tomography (CT) scanners, and ventilators, to LED lighting, charging, displaying, “internet of things (IoT)”, infrastructure, and aerospace. In these areas, miniaturization is in progress in regard to device dimensions and electronics [1,2]. For instance, this is the case for smartphones, wearable devices, and tablets. Miniaturization poses several challenges; one of these challenges is the processing and drilling of smaller holes into stronger PCBs [3].

A printed circuit board comprises a thin base board made of resin-bonded paper or fiberglass (insulator). This baseboard is coated at one or both sides with a thin layer of copper (conductor), which has been printed on the board into specific circuits. Components need to be attached to the board, which necessitates drilling holes into the PCB at designated locations. To obtain high reliability, these holes should be round, homogeneous, and without debris (i.e., burrs) [1]. Drilling of these materials has become challenging [3], as the materials for printed circuit boards have moved toward lightweight high-strength materials [4], resulting in higher wear of cutting and drilling tools during machining [5]. Further, the smaller hole diameters (and smaller microdrill diameters) lead to exacerbation of microdrill failure, often due to fracture of the microdrill at the joint between the drill shank and the drill body (flute) due to stress concentration [6]. To alleviate the issue of accelerated wear, highly wear-resistant and high-strength materials, such as WC-Co, have been established as cutting and drilling tools [7]. To improve durability and wear resistance further and minimize build-up edge, diamond coatings can be applied to these tools [8], and commercial diamond-coated cutting and drilling tools are available. However, the growth of diamonds on WC-Co necessitates appropriate pretreatment, seeding procedures, and specific growth parameters [9,10,11].

The pretreatment is necessary as Co at the surface catalyzes the generation of graphite instead of diamond, resulting in deterioration of film adhesion and poor diamond quality [10,12]. A general pretreatment to avoid this is the application of Murakami solution, a base that removes WC from the surface, roughening it, followed by treatment with Caro’s acid, a solution that dissolves the cobalt from the surface. This process was patented more than 30 years ago [13] and has been optimized for general cutting and drilling tools. Other approaches are acid-base-acid etching in combination with interlayers or diffusion barriers [14]. Problems arise when the cutting and drilling tools are miniaturized, as the surface-to-volume ratio increases and the aforementioned etching decreases the fracture strength of the composite hard metal due to “excessive” removal of the cobalt binder [15]. Therefore, researchers have investigated the optimal pretreatment parameters (etching) for WC-Co microdrills [16]. For instance, Geng et al. [15] showed for a 0.5 mm diameter microdrill (WC-Co, 12 wt% Co) that only 3 min etching with Murakami solution is enough to etch sufficient WC from the surface, further etching did not lead to higher wt% of Co on the surface. The subsequent acid etching yielded a “Co depletion layer” of 6.5 µm after 15 s, and this layer would grow up to 15 µm after 120 s. Simultaneous with the formation of the Co depletion layer, the fracture strength of the hard metal declines. Therefore, a balance between etching length for Co depletion and fracture strength has to be found.

After the pretreatment, the WC-Co needs to be seeded with a nanodiamond seeding solution with advantageous colloidal stability [17], as the seeding density affects the adhesion of the diamond coating [18]. Subsequently, the growth process is of utmost importance for the quality and adhesion of the diamond coating as well as the performance in cutting or drilling operations. For WC-Co, diamond coating adhesion is, in general, weak due to high residual stress stemming from the mismatch in thermal expansion coefficient between the WC-Co substrate and diamond [19,20]. This stress is compressive, leading to spallation of the diamond coating upon cutting or drilling due to stress concentration (at the WC-Co/diamond interface) [21]. The build-up of thermal stresses is inevitable due to the temperatures of chemical vapor deposition (CVD) diamond growth, albeit low-temperature diamond growth with poor diamond quality is possible [22]. In this regard, interlayers can reduce or mitigate thermal stresses that originate from thermal mismatch. Interlayers with good interfacial bonding, high strength, and a thermal expansion coefficient (COE) between diamond and hard metal are used for this purpose. Examples of such interlayers are TiAlN [23], TiC and/or TiN [24], SiC [25,26], Al-Al_2_O_3_ [27], Al-AlN [27], Cr/CrN/Cr [20,28], Cr_2_O_3_-Cr [29], CoB, TiMoTa [30], and multilayer TiN/Al_2_O_3_/TiCN [12,23,31,32]. The SiC phase features a distinct advantage; namely, it can be grown in the hot filament chemical vapor deposition (HFCVD) chamber at the same time as diamond is grown by introducing a Si source (i.e., tetramethyl silane, TMS) into the reactive gas mixture [33]. The result is a SiC/diamond composite, for which the content of diamond (or SiC) can be adjusted by the flow rate of the Si source (or the concentration of the Si source in the gas phase) or by the distance of the substrate from the filaments (temperature) [34,35,36]. This control over the interlayer coating composition affords the ability to tailor the residual stress of interlayers based on SiC/diamond composites and the associated adhesion and maximize wear resistance. A further advantage is the fact that a pure diamond coating can be directly grown on the SiC/diamond composite coating, as the diamond grows on this interlayer without an intermittent seeding step.

The surface finish of a diamond coating has a grave influence on the cutting and drilling performance. Though it was shown that microcrystalline diamond coatings feature higher wear resistance [37], the higher roughness of these coatings [38] and the higher friction coefficient (COF) [39] lead to perceived poorer performance of such coatings compared to nanocrystalline coatings. Therefore, it appears favorable to deposit a nanocrystalline diamond top layer on cutting tools in spite of the poorer overall wear resistance compared to the microcrystalline counterpart. A further advantage of this kind of bilayer coating with a nanocrystalline top layer is the ability to mitigate crack propagation often observed in microcrystalline coatings [40,41].

In this report, WC-Co microdrills with diameters of 0.8 and 0.125 mm were used as starting material for the deposition of diamond coatings and subsequent wear resistance tests (drilling of PCBs). Initially, the pretreatment with Murakami solution and Caro’s acid was scrutinized and optimized toward a balance between the Co depletion layer and fracture strength of the hard metal. Then, a SiC/microcrystalline diamond interlayer was deposited. Finally, the WC-Co microdrills coated with the interlayer were outfitted with a nanocrystalline diamond top layer. The performance of this composite coating was evaluated against nanocrystalline and microcrystalline diamond coatings deposited directly after pretreatment on the WC-Co substrate, i.e., by Rockwell C indentation and by drilling experiments, showing the wear and spallation of the diamond coatings.

## 2. Experimental Section

### 2.1. Materials

Flat WC-Co substrates with Co content of ca. 6 wt% (size 10 × 10 mm, model number YG6X4130511) were purchased from Zhuzhou Diamond Cutting Tools Co. (Zhuzhou, China). The microdrills (model number A129QV) with diameters of 0.8 mm (6 wt% Co) and 0.125 mm (8 wt% Co) were supplied by Shenzhen Jinzhou Precision Technology Co., Ltd., Shenzhen, China. Both types of microdrills have a helix angle of 35 ± 2°.

Potassium hydroxide (AR), potassium ferrocyanide (AR), and sodium dodecylbenzenesulfonate (SDBS, AR) were purchased from Aladdin (Shanghai, China). AR denotes the analytical reagent grade of the chemicals. Hydrogen peroxide aqueous solution (30 wt%) was purchased from Shanghai Lingfeng Chemical Reagent Company (Shanghai, China). Sulfuric acid (AR) (98 wt%) and nitric acid (AR) (65 wt%) were obtained from Dongguan Dongjiang Chemical Reagent Company (Dongguan, China), and detonation nanodiamond (DND) powder with a diameter of 100–110 nm was obtained from Shenzhen Tongli Micro Nano Technology Co., Ltd. (Shenzhen, China). The hydrodynamic diameter and Zetapotential determined by dynamic light scattering (Zetasizer Nano ZS, Malvern Panalytical Ltd., Malvern, UK) were 124 nm and −38.9 mV, respectively.

### 2.2. Etching Process of the Cemented Carbide Substrates

Initially, the Murakami solution was prepared by adding 10 g potassium hexacyanoferrate and 10 g potassium hydroxide in 100 mL DI water. The WC-Co substrates were immersed in the alkaline Murakami solution for varying etching times (between 0 and 20 min) while being agitated ultrasonically. Subsequently, the etched samples were scrutinized by scanning electron microscopy (SEM, for morphology) and energy dispersive X-ray spectroscopy (EDS, for composition).

**Figure 1:**
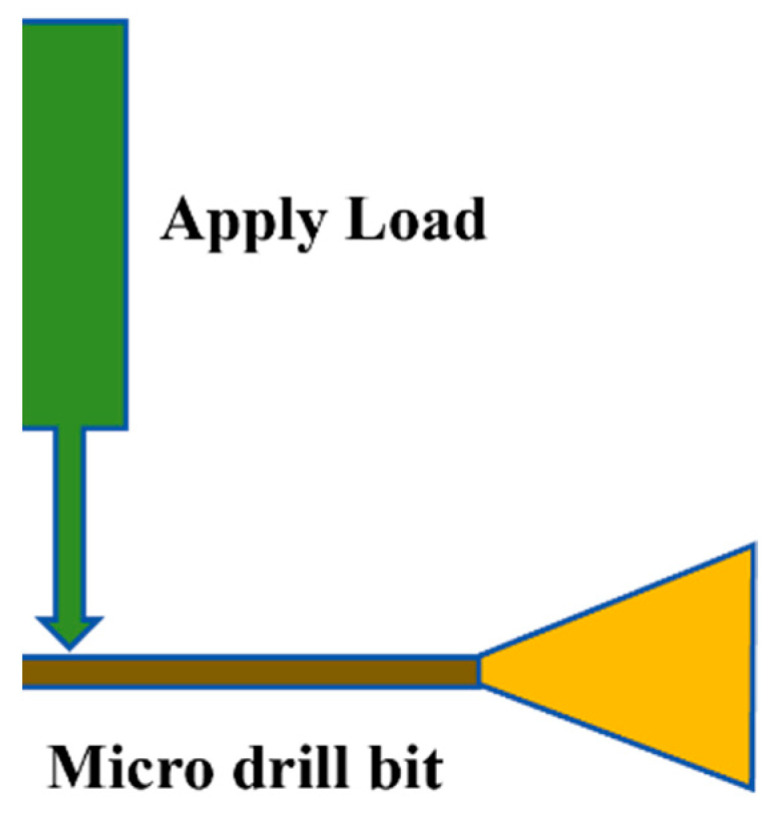
Measurement setup for determination of stress–strain curves and fracture strength of microdrills.

Similarly, the etching of microdrills with diameters of 0.800 mm and 0.125 mm were carried out. Etching with the alkaline Murakami solution was carried out for 3, 6, or 9 min, followed by acid etching for 15 s. The acidic etchant was prepared by mixing 10 mL of concentrated sulfuric acid with 100 mL of hydrogen peroxide. The effect of the alkaline etching time on the relative fracture strength of the microdrills was subsequently scrutinized. A microdrill was placed in a test rig, where it was fixed at the drill shank. Force was applied at the flute of the microdrill, leading to a displacement (bending of the flute) of the microdrill (a scheme of the experiment is depicted in Figure 1). Force versus displacement curves were captured, and the critical load at fracture was noted for the microdrills.

### 2.3. Nanodiamond Seed Preparation and Seeding

0.1 g of DND powder and 2 g of sodium chloride were balanced and added into a ductile iron tank for ball milling with a planetary ball mill. The ball milling was conducted at 350 rpm for 180 min. After ball milling, 80 mL of concentrated nitric acid was added, and the colloid was stirred for 3 h. Afterward, the oxidized nanodiamond samples were taken out and centrifuged three times at 7000 rpm for 8 min. After each centrifugation step, the supernatant was removed, and 30 mL of deionized water was added. After centrifugation, 100 mL 1 × 10^−6^ mol/L sodium dodecylbenzenesulfonate aqueous solution was added, and the pH of the prepared colloidal solution was adjusted to 10. The hard metal samples were seeded ultrasonically with this solution for 15 min, followed by carefully rinsing with deionized water and blow drying with nitrogen.

### 2.4. Preparation of the Diamond Coatings on Microdrills

The microdrills were etched with Murakami solution for 3 min, followed by acid etching for 15 s. Then, the microdrills were seeded ultrasonically with the nanodiamond colloid. Afterward, the three different diamond coatings were grown. Nine filaments made of tungsten were aligned horizontally, and the microdrills were aligned vertically. The deposition parameters are listed in Table 1. The pressure during the chemical vapor deposition process (CVD) was set to 1500 Pa. The final coating thickness was ca. 2.5 µm as this thickness was suggested to yield a good compromise between the adhesion strength of the coating and wear resistance [42].

After deposition, the morphology and composition were characterized by SEM, XRD, and Raman. Raman spectra were recorded with the HORIBA LabRAM HR800 Evolution Raman spectrometer (HORIBA Trading Co., Shanghai, China) using an excitation wavelength of 633 nm. The surface morphology of the deposited diamond films was analyzed by field emission scanning electron microscopy (FE-SEM, Hitachi, S-4800, Tokyo, Japan). The accelerating voltage was 5 kV. X-ray diffraction (XRD) patterns were taken in a θ/2θ geometry (Rigaku MiniFlex 600 X-ray, Cu Kα radiation, Tokyo, Japan) with a scan rate of 8° min^−1^. Rockwell C indentation was conducted on the flat diamond-coated samples at an applied indentation force of 1470 N.

### 2.5. Performance of the Diamond Coatings on Microdrills

The effectiveness of the diamond-coated microdrills (performance) was tested for drilling holes in printed circuit boards (PCBs). The PCBs were either S1000-2M (Shengyi Technology Company, Dongguan, China) or HL-832NSF (Mitsubishi Gas Chemical Company, Tokyo, Japan) for drilling with the 0.8 mm or 0.125 mm microdrill, respectively. The composition of the S1000-2M is continuous filament fiberglass, copper, brominated epoxy resin, and inorganic filler. The HL-832NSF is a bismaleimide-triazine (BT) resin. For drilling, the Hitachi ND-6Y220E high-speed drilling machine was used. The drilling parameters for the 0.80 mm diameter microdrills were 65 krpm, drop speed 40 mm/s, and PCB (S1000-2M) thickness 0.80 mm. The drilling parameters for the 0.125 mm diameter microdrill were 180 krpm, drop speed 25 mm/s, and PCB (HL-832NSF) thickness 0.30 mm. The generated holes and the wear (i.e., flank wear) at the microdrills were inspected by optical microscopy.

## 3. Results

### 3.1. Fracture Strength of Pretreated Microdrills

**Figure 2:**
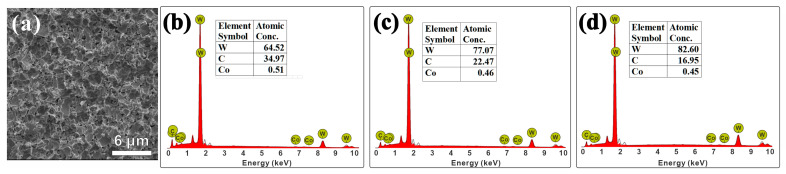
SEM morphology of a 0.125 mm WC-Co micro drill after Murakami etching for (**a**) 3 min, followed by acid etching for 15 s. EDS spectra for the samples etched with Murakami solution for (**b**) 3 min, (**c**) 6 min, (**d**) 9 min, followed by acid etching for 15 s.

Initially, pretreatment was investigated on flat WC/Co substrate with ca. 6 at% Co to ascertain the depletion of Co. SEM micrographs before and after treatment with Murakami solution (5–15 min) and Caro’s acid is discussed in the Supporting Information Figures S1 and S2, respectively. During the pretreatment process, the microdrills were etched by Murakami solution, followed by Caro’s acid. To optimize the etching process, the Murakami etching time was varied from 3 to 9 min while the acid etching time was maintained at 15 s. Figure 2a shows the surface morphology of the etched (3 min Murakami and 15 s Caro’s acid) WC-Co microdrill, while Figure 2b–d show the cobalt depletion at the surface of the 0.125 mm microdrills measured by EDS after etching. Figure 2a shows that after acid etching, the cobalt element between WC particles is removed. Similar results are observed for the microdrills etched for 6 and 9 min with Murakami solution followed by Caro’s acid etching for 15 s, as shown in Figure S3. Figure 2b–d shows that the cobalt content at the surface of the WC-Co microdrills was depleted to 0.51% (3 min Murakami), 0.46% (6 min Murakami), and 0.45% (9 min Murakami). Therefore, a Murakami etching time of 3 min is sufficient. Subsequently, the strength after treatment with different alkali corrosion times was evaluated to find the alkali corrosion time that could ensure optimal operation of the microdrills.

**Figure 3:**
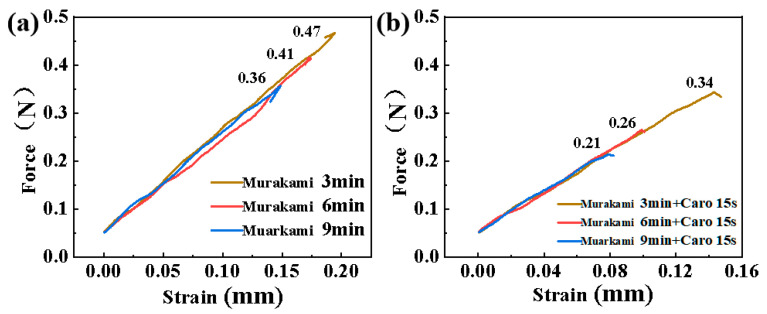
Force-strain curve of micro-drills (0.125 mm) after etching with (**a**) Murakami solution for 3 to 9 min and (**b**) Murakami solution (different etching times) and Caro’s acid for 15 s.

Figure 3 shows the load–displacement curve of the microdrills with a diameter of 0.125 mm after treatment with different etching times. The load at fracture of the microdrills decreased with the increase in alkali etching time, which is consistent with etching and embrittlement due to the removal of the WC phase observed previously [15]. Figure 3b shows the load at fracture of the microdrill decreased further for the process of alkali etching, followed by acid etching for 15 s. Notably, the effect of short-term acid etching with Caro’s acid on the load at fracture of the micro drills is far greater than that of etching with Murakami solution. The etching time with Caro’s acid was not varied as the cobalt depletion layer has been established already after the short etching time of 15 s, and there is virtually no change in fracture strength for longer etching times (see ref. [15]).

The etched microdrills were evaluated for their stability during normal operation (drilling). The gist of this evaluation is that only the microdrills treated with Murakami solution for the shortest time (3 min) did not show failure, i.e., fracture of the microdrill at the drill shank. Longer Murakami leaching times of > 3 min led to the microdrill’s failure during normal operation, as detailed in Table 2. This failure of the microdrills during PCB drilling is a common problem of filigree diamond-coated WC-Co microdrills [43], and optimization of the etching process is thus of utmost importance for diamond deposition and the use of microdrills.

### 3.2. Deposition of Micro, Nano, and Composite Intermediate Layer Coatings

**Figure 4:**
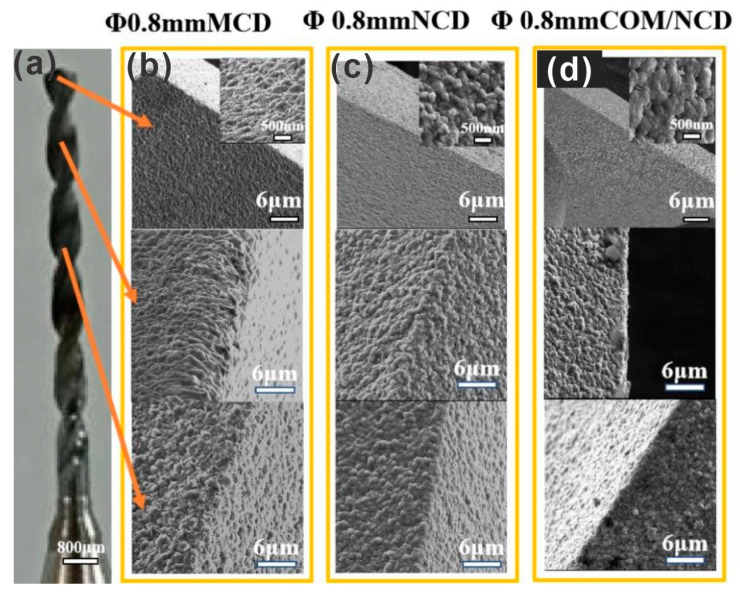
Growth of diamond on WC-Co microdrills (pretreatment 3 min Murakami, 15 s acid). (**a**) Optical image of a microdrill with a diameter of 0.8 mm. SEM micrographs of the diamond coatings along the microdrill (cutting edge, ca. 1.25 mm below the cutting edge, and ca. 4 mm below the cutting edge). SEM micrographs for (**b**) the microdiamond, (**c**) nanodiamond, and (**d**) SiC/nanodiamond coating on the WC-Co microdrill.

**Figure 5:**
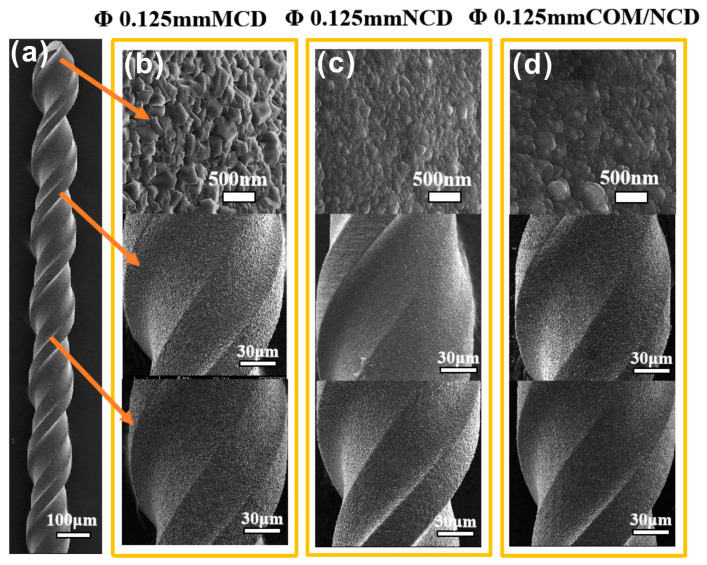
Growth of diamond on WC-Co microdrills (pretreatment 3 min Murakami, 15 s acid). (**a**) SEM of a microdrill with a diameter of 0.125 mm. SEM micrographs of the diamond coatings along the microdrill (cutting edge, ca. 0.4 mm below the cutting edge, and ca. 0.8 mm below the cutting edge). SEM micrographs for (**b**) the microdiamond, (**c**) nanodiamond, and (**d**) SiC/nanodiamond coating on the WC-Co microdrill.

After optimization of the pretreatment for the microdrills with 0.8 mm (Figure 4) and 0.125 mm (Figure 5), three different diamond films were coated on the microdrills, namely, microcrystalline, nanocrystalline, and composite coating (diamond/SiC interlayer and nanocrystalline diamond top layer). The reason for the composite interlayer, as well as the analysis and optimization thereof, are discussed in the Supporting Information. The accompanying Figures S3–S5 describe the optimization of the composite microcrystalline diamond/SiC interlayer on flat WC/Co (6 at%) substrates. Figure S4 shows the SEM morphology of the resulting composite layer at different gas compositions. Figure S5 shows the morphology of the optimized composite layer, the cross-section of the composite layer, and the C (diamond) and Si (SiC) distribution by EDS mapping. Figure S6 shows the XRD pattern of the composite coating, signifying the presence of SiC, WC, and diamond. Further, morphology and cross-sectional SEM micrographs of the three different coatings (microcrystalline, nanocrystalline, and composite interlayer with nanodiamond finish) are shown in Figure S7, indicating an overall thickness of ca. 2.5 µm of all the coatings. Specifically, the thickness of the microcrystalline, nanocrystalline, and composite (two-layer) coating was 2.4 ± 0.4 µm, 2.5 ± 0.1 µm, and 2.4 ± 1.6, respectively, indicating a higher thickness deviation for coatings with microcrystalline diamond. The microdrills were coated from the cutting tip down ¾ of the drill body toward the drill shank. Figure 4 shows diamond-coated microdrills (0.8 mm diameter) with the microcrystalline (MCD), nanocrystalline (NCD), and diamond/SiC composite interlayer + nanodiamond top layer (COM/NCD). The SEM micrographs taken from the flank face and chisel edge show a homogeneous coating for all diamond coatings. Similarly, homogeneous diamond coatings were found for micrographs taken 1/8 and 1/3 down the microdrill body. Similar results were obtained for the 0.125 mm diameter microdrill shown in Figure 5.

**Figure 6:**
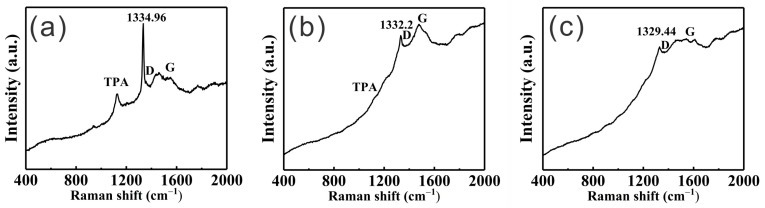
Raman spectra of (**a**) the microdiamond coating, (**b**) the nanodiamond coating, and (**c**) the diamond/SiC composite interlayer + nanodiamond top layer.

Figure 6 shows the Raman spectra of the three coatings shown in Figure S7. Figure 6a is the Raman spectrum of the microcrystalline diamond coating, which features a prominent peak at 1334.96 cm^−1^, denoting the presence of diamond. The peak is sharp, signifying the high quality of the diamond that has grown. However, the peak is shifted away from the general diamond peak at 1332 cm^−1^ due to thermal stresses originating from a mismatch in thermal expansion coefficient and intrinsic stresses. The average residual compressive stress is −1.55 GPa, calculated by $\;\sigma =-0.526*\left(v_{b}-v\right)\;GPa$ [44], where *v*_b_ and *v* are the peak location of the diamond peak in a stressed state and the unstressed peak location in Raman spectroscopy, respectively. This relatively high compressive stress may lead to spallation of the coating under external stresses (i.e., cutting or drilling). Other features in the Raman spectrum are the peak for trans polyacetylene (TPA) and the D and G bands of graphene. Similarly, Figure 6b shows the Raman spectrum of the nanocrystalline coating with a broadened peak at 1332.2 cm^−1^. The peak broadening is a common occurrence for nanocrystalline coatings due to a higher abundance of defects in the diamond crystallites. The average calculated compressive stress is −0.11 GPa, lower than for the microcrystalline coating due to the higher abundance of grain boundaries (intrinsic stress build-up, which is often tensile—believed to stem from snapping together of the diamond grains during growth, counteracting the compressive thermal stress). The diamond peak is weak compared to the graphite G band due to the higher sensitivity of the G band in Raman [45]. However, this indicates a higher abundance of graphite generated during the CVD diamond growth, likely located at the grain boundaries of the nanocrystalline diamond. Finally, Figure 6c depicts the Raman spectrum of the composite coating (diamond/SiC interlayer + nanodiamond top layer). The spectrum is similar to the nanocrystalline diamond spectrum, except for the peak location of the diamond Raman peak, which is shifted 2.5 cm^−1^ towards tensile stress (+1.34 GPa). As stated before, the tensile stress stems from the growth process (snap-in) of diamond crystallites [46,47]. In the case of the composite film, little compressive stress is built up at the WC-Co/composite interlayer interface due to a mismatch in the thermal expansion coefficient. Therefore, the tensile stress (intrinsic stress) of the secondary (nanodiamond) layer predominantly contributes to the overall stress observed. It should be noted that the stress is an average stress. Stresses are different at the interface of the cemented carbide with diamond (or the interlayer) and inside the diamond film itself and depend on many factors (i.e., grain size, doping, and other coating parameters) [48].

**Figure 7:**
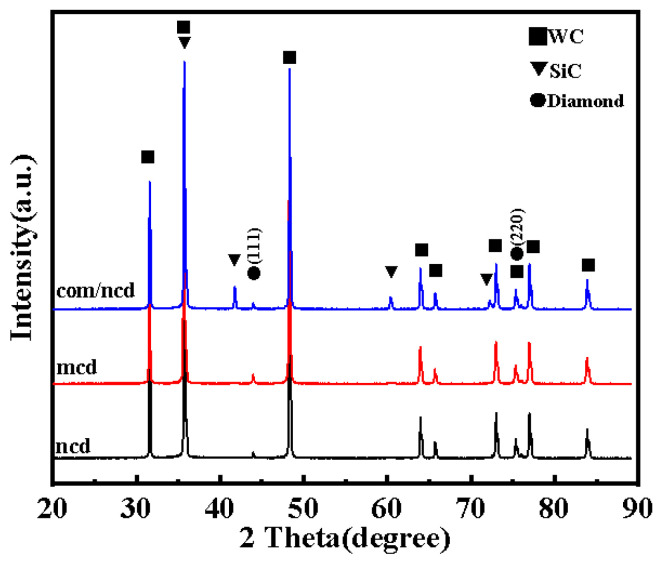
XRD patterns of the microcrystalline diamond coating, nanocrystalline diamond coating, and composite interlayer + nanodiamond top layer.

Figure 7 shows the XRD patterns of the three coatings, signifying the phase composition of the coatings. The different coatings are denoted ncd, mcd, and com/ncd for the nanocrystalline diamond, microcrystalline diamond, and diamond/SiC composite coating with nanocrystalline diamond top layer. Since the diffraction thickness of XRD is greater than the diamond coating thickness of 2–3 µm, diffraction patterns of the WC-Co matrix are detected beside the diffraction patterns for the diamond crystallites. Diffraction peaks at 2 theta of 75.3° and 43.9° signify the diamond (220) and (111) crystal lattice planes, respectively. The diffraction peaks at 59.72° and 71.4° denoting β-SiC (220) and (311) lattice planes, respectively, are only observed for the composite coating. The diffraction peaks of the SiC (111) plane and the diamond (220) plane at 35.3° and 75.2°, respectively, overlap with the diffraction peaks of the (100) plane and the (200) plane of the WC alloy and cannot be discerned here.

**Figure 8:**
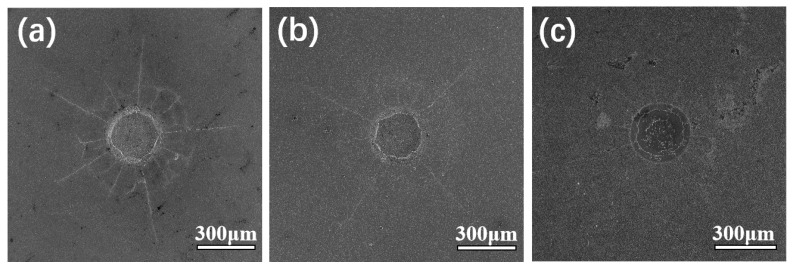
Indentation craters of (**a**) microdiamond coating, (**b**) nanodiamond coating, and (**c**) diamond/SiC composite interlayer + nanodiamond top layer after Rockwell C indentation with a force of 1470 N. The coating is located on substrates of the flat WC-Co (pretreated with 3 min Murakami, 15 s acid).

To evaluate the film adhesion, Rockwell C indentation tests were carried out on the three different coatings, which were deposited on flat WC-Co substrates. Figure 8 shows indentation craters after Rockwell C indentation with a load of 1470 N of the diamond-coated WC-Co substrates. No spallation or debris formation has been observed. The diamond remained attached to the WC-Co hard metal for all diamond coatings. In lieu of a difference in detachment of diamond coating debris, crack propagation, and crack length are indicators for adhesion strength of diamond coatings (see also: VDI 3198 indentation test [41]) [9,49,50]. When considering radial crack lengths, the number of smaller cracks, and the existence of a clearly visible circular crack in the vicinity of the indentation crater, the poorest adhesion is observed for the microcrystalline diamond coating, which correlates with the compressive residual stress and issues with crack propagation in microcrystalline coatings due to the columnar growth of the crystallites [51]. The single-layer nanodiamond coating shows improved adhesion, as only four larger cracks and a few barely visible shorter cracks are visible. Still, the crack propagation (radial cracks) is rather large. The best adhesion due to the presence of the shortest radial cracks was observed for the composite/nanodiamond double-layer coating. The crack propagation zone is also small, indicating that the adhesion of the diamond coating can be increased by using the composite layer as a transition layer.

### 3.3. Performance of the Micro, Nano, and Composite Diamond Coatings for Drilling PCBs

During the drilling of PCBs, microdrills are expected to experience wear. Further, poor adhesion of diamond coatings may lead to flaking and peeling of diamond coatings, which may be exacerbated due to crack propagation. Therefore, the coating adhesion to the microdrills and drilling performance were investigated. The drilling parameters and the PCB material are delineated in the experimental section.

**Figure 9:**
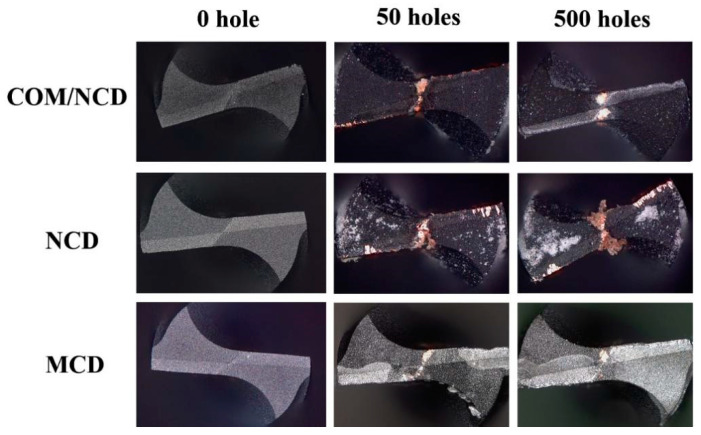
Surface morphology of the diamond-coated microdrills (diameter 0.8 mm) after drilling 50 and 500 holes in the PCB. Drilling parameters: 65 krpm, drop speed 40 mm/s, and PCB thickness 0.80 mm.

Figure 9 shows optical microscopy images of the three different diamond coatings before drilling (0 holes) and after drilling 50 and 500 holes in the PCB. Similar to the SEM micrograph in Figure 4, the initial vertical view optical microscopy image of the drill tip shows homogeneous diamond coatings for all types of diamond coatings. After 50 drilled holes, brighter areas on the cutting tip at the flank face (at and near the cutting edge) can be discerned for the microdiamond coating. These brighter areas denote the peeling of the microcrystalline coating due to cutting-induced stress (compressive) and the residual compressive stress, resulting in weak adhesion of this diamond coating. Peeling of the microcrystalline diamond coating is exacerbated with an increased number of holes drilled, and after 500 drilled holes, most microcrystalline diamond is peeled off the flank faces of the drill tip. Further, strong wear can be observed at the cutting edge as well as the chisel of the drill tip. Slight build-up edges can be observed for this type of coated microdrill. Build-up edge (*Aufbauschneide*) is a common phenomenon observed in the drilling and cutting of metals with hard metals that can be avoided by low-friction materials like diamonds.

The nanodiamond coating has been suggested to feature better adhesion, albeit wear resistance is lower compared to microdiamond coatings. After 50 holes were drilled with the nanocrystalline diamond-coated drills, the optical image of the drill tip shows several small and bright areas at the flank face of the drill, denoting areas where the diamond coating flaked off, yet no large area peeling of the coating is observed due to the lower crack propagation of nanocrystalline diamond coatings compared to microcrystalline coatings. Further, coppers sticking at the chisel and the cutting edges (build-up edges) can be observed. The build-up edge is sometimes associated with a shortened tool lifetime and poor drilling performance [52]. After 500 holes, several large peeled areas are observed, and the build-up edge is exacerbated, signifying a decline in the coating’s performance. Peeling of the nanodiamond coating might stem from compressive residual stress at the substrate/nanodiamond interface induced by thermal stresses, akin to the microcrystalline coating (even though the average residual stress of the coating is only slightly compressive).

Compared to these findings, the composite coating (diamond/SiC interlayer & nanodiamond top layer) shows improved drilling performance and tool lifetime. After 50 drilled holes, no flaking or peeling of the composite coating was observed, except for the chisel area. At the chisel, a bit of copper also adhered due to the bare metal matrix. The cutting edge appears sharp with a minute adhesion of copper from the PCB (minute build-up edges). After drilling 500 holes, the cutting edge’s color became brighter, indicating the wear of the nanocrystalline diamond coating. Still, neither peeling of the composite coating at the cutting edge nor at the flank face was observed. Little build-up edges can be discerned. Further, the area at and near the chisel without the diamond coating has grown only slightly, indicating good adhesion and performance of the composite coating compared to the micro- or nanocrystalline coatings discussed before.

**Figure 10:**
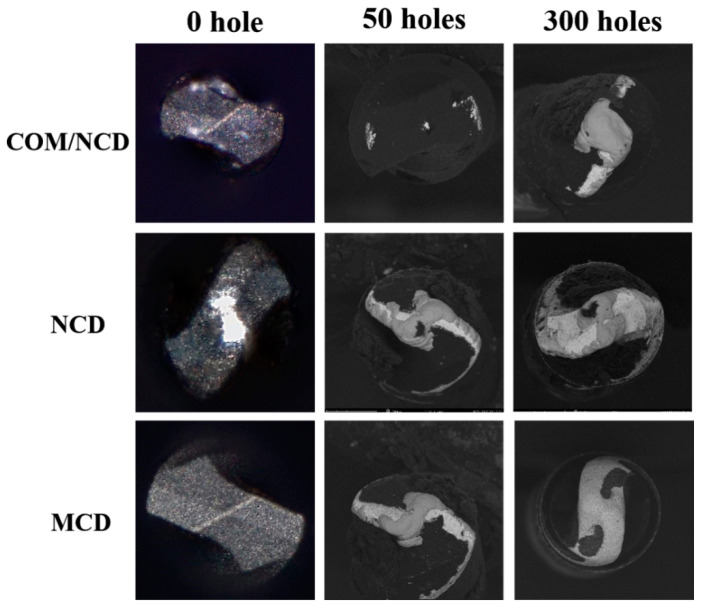
Surface morphology of the diamond-coated microdrills (diameter 0.125 mm) after drilling 50 and 300 holes in the PCB. Drilling parameters: 180 krpm, drop speed 25 mm/s, and PCB thickness 0.3 mm.

Figure 10 shows the drill tip of diamond-coated microdrills with a diameter of 0.125 mm before, during, and after drilling 300 holes in PCB. In contrast to the measurement in Figure 9, the drill tip was measured with SEM to improve visibility and clarity of peeling areas and wear on the drill tip of the microdrills. Again, the microdiamond coating performs poorly, with flaking and peeling of a wide region of the coating during the drilling of the first 50 holes. The peeling of the coating is concentrated near the cutting edge at the flake face (due to stress concentration). Minor peeling and wear are observed at the minor cutting edge. Build-up edges are observed at the chisel region of the microdrill. Delamination of the microcrystalline diamond film is exacerbated after 300 drilled holes, with the diamond coating peeled from the better part of the flake face and the chisel area. For the nanodiamond coating, a similar wear pattern is observed, albeit the build-up edge is severer.

In contrast to the observed delamination of the single layer diamond coatings from the 0.125 mm diameter microdrill, wear and delamination of the diamond coating is starkly reduced for the composite coated microdrill after drilling 50 holes in the PCB. Obviously, no delamination was observed after drilling 50 holes, while wear areas at the flake face in the vicinity of the minor cutting edge can be discerned. Similarly, wear is observed in the chisel area (highly localized). Wear at the cutting edge or other flake face regions cannot be discerned. After 300 drilled holes with this (composite) diamond-coated microdrill, wear is observed at the cutting edge and flake face, but no delamination of the coating is observed. Further, the bright region in the chisel region denotes the adhesion of copper to the microdrill. Therefore, the composite coating exhibited the best wear resistance and coating adhesion of the three coatings discussed in this article. It is important to note that both sizes of coated microdrills did not fail due to fractures of the drill body from the drill shank, which can be a problem for microdrills after the treatment with Murakami solution.

**Figure 11:**
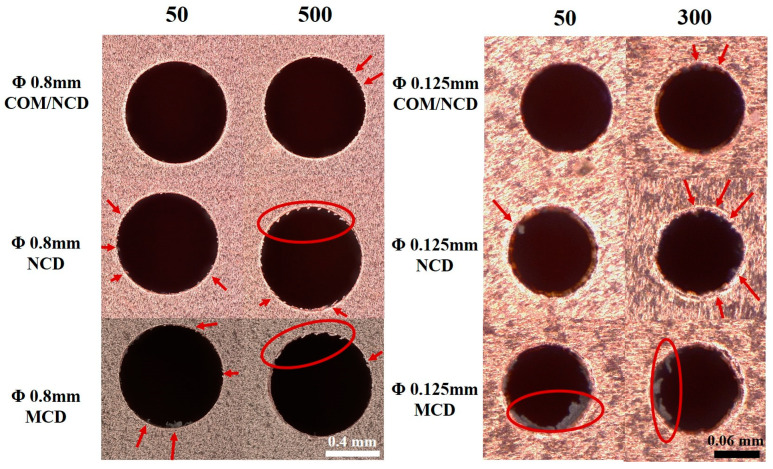
Appearances of the holes drilled in PCB after drilling 50 and 500 holes with the same microdrill measured by optical microscopy. The diameter of the diamond-coated microdrills were (**left**) 0.8 mm and (**right**) 0.125 mm. The red arrows highlight the formation of burrs. The microdrills were prepared with the optimized pretreatment, and the composite coating was made using the optimized TMS flow rate.

Figure 11 shows the appearance of the holes, which were drilled with the three different diamond-coated microdrills (either 0.8 or 0.125 mm in diameter). First, the holes obtained with the larger microdrill are discussed. Some burrs (red arrows) and some debris/poorly cut chips are observed for the 50th hole drilled with the microcrystalline diamond-coated microdrill. After the 500th hole, extensive burr formation is observed, and the drilled holes are slightly ovoid. The poor drilling performance stems from poor adhesion and wear resistance of the microcrystalline diamond coating on the drilling tool, as the coating was already peeled after 50 holes, exacerbated after 500 holes, resulting in build-up edges. A similar result is observed for the nanocrystalline diamond coating on the microdrill, albeit the drilling performance is better after only 50 holes. A few burrs can be observed here. However, after drilling 500 holes, burr formation is similar to that of the microcrystalline coating. In contrast, the composite-coated microdrill exemplifies improved drilling performance after 50 and 500 drilled holes. After 50 holes, no burrs can be discerned. Further, burr formation after 500 drilled holes is minimal. This improved performance is a direct result of the low friction of diamonds (build-up edges are less likely) and the good adhesion of the composite coating (no delamination of the coating).

Generation of filigree diamond-coated microdrills is difficult, and their stability and diamond-coating adhesion are often limited. Therefore, microdrills with a diameter of 0.125 mm were coated using the same procedure with the three different diamond coatings, and then, the drill performance of the microdrills was investigated. For both the microcrystalline and nanocrystalline diamond-coated microdrills, poor drilling performance was observed already after drilling 50 holes, as burrs and debris are clearly visible. Burr and debris generation is exacerbated after 300 drilled holes, while at this point, the hole gets irregular (non-circular), signifying poor drilling performance. The poor drilling performance of these two coatings stems from the delamination of both coatings from the microdrill already after 50 drilled holes, as shown in Figure 10, and the loss of protection against the build-up edge. In contrast, the composite-coated microdrills perform far better. The hole drilled after using the same drill for drilling 50 holes features a high circularity, and no burrs or debris can be discerned. Even after 300 drilled holes, the generated hole is circular with only a few minute burrs. The improved drilling performance stems from the improved adhesion of the composite film in combination with the excellent tribological properties of the diamond coating (i.e., low friction) and minimization of build-up edges. Therefore, the composite diamond-coated microdrill shows superior drilling performance. Note that the coated microdrills did not fracture during this experiment due to the optimization (or compromise between) fracture strength of the metal matrix and pretreatment time for subsequent diamond growth.

## 4. Conclusions

For microdrills and other filigree cutting and drilling tools, a balance between the etching duration for removal of Co from the surface of the WC/Co metal matrix and the fracture strength of the metal matrix has to be struck. Therefore, the effect of etching time on the cobalt content of the WC cemented carbide matrix in combination with the fracture strength was studied, as well as the adhesion of diamond coatings after optimizing the pretreatment parameters. The cobalt content on the surface of the alloy continued to increase with prolonged Murakami etching. This increase effectively enhances the subsequent cobalt removal by the Caro’s acid. For microdrills, the optimized etching parameters were 3 min Murakami etching and 15 s Caro’s acid etching, ensuring a low enough cobalt content at the surface for high-quality diamond growth while maintaining a high enough fracture strength to mitigate fracture of the microdrills during operation. Secondly, three different diamond coatings were deposited on the etched substrates, namely, microcrystalline, nanocrystalline, and a composite comprising a diamond/SiC composite interlayer and a nanocrystalline top layer. The bilayer composite coating featured the best adhesion on flat WC-Co substrates as well as on the microdrills (diameter 0.8 and 0.125 mm). Further, the composite-coated microdrills feature improved performance for drilling of PCB compared to the single-layer diamond coatings, which stems from the improved coating adhesion due to lowered thermal stresses at the substrate coating interface, lower wear of the microdrills, and the low friction coefficient of nanodiamond finish, resulting in improved drilling performance and higher hole quality.

## Figures and Tables

**Table 1:** Deposition parameters used for depositing the three different diamond coatings on the microdrills.

Coating Type	H_2_ (sccm)	CH_4_ (sccm)	TMS (sccm)	Filament-Microdrill Distance (mm)	Argon (sccm)	Deposition Time (h)
Microdiamond	800	32	0	23	0	1.5 h
Nanodiamond	800	88	0	25	500	3 h
Dia/SiC interlayer + nanodia. top layer	800	(Interlayer) 32 *	80 *	23 *	0 *	1 h *
		(top layer) 88 ^#^	0 ^#^	25 ^#^	500 ^#^	2 h ^#^

* deposition parameters for the diamond/SiC interlayer deposition prior to nanodiamond growth. ^#^ deposition parameters for nanodiamond top layer growth.

**Table 2:** Microdrill (0.125 mm diameter) fracture with different corrosion parameters.

Murakami Etching Time (min)	Acid Etching Time (s)	Fracture Load (N)	Failure of the Microdrill During Drilling of PCBs
3	15	0.34	No
6	15	0.26	Yes
9	15	0.21	Yes

## Data Availability

The original contributions presented in this study are included in the article/Supplementary Materials. Further inquiries can be directed to the corresponding authors.

## Supplementary Materials

The following supporting information can be downloaded at https://www.mdpi.com/article/10.3390/ma17225593/s1, Figure S1: (a) SEM surface morphology of as-received WC-Co substrate. (b) EDS spectrum of as-received WC-Co substrate. Figure S2: The surface morphology of the WC-Co substrate after etching for (a) 5 min, (b) 10 min, (c) 15 min, and (d) 20 min with Murakami solution. Below the SEM micrographs the corresponding EDS spectra are shown. (e) Composition of the etched WC-Co substrates determined by EDS. Figure S3: SEM morphology of a 0.125 mm WC-Co microdrill after Murakami etching for (a) 6 and (b) 9 min, followed by acid etching for 15 s. Figure S4: Surface morphology of diamond/silicon carbide composite films deposited on flat WC-Co sample after 1. etching for 15 min with Murakami and 15 s with Caro’s acid and 2. seeding with milled DNDs. The TMS flow rates were (a) 20 sccm, (b) 40 sccm, (c) 60 sccm, (d) 80 sccm, (e) and 100 sccm. Figure S5: Surface morphology of diamond/SiC composite films made with 80 sccm TMS on flat WC-Co substrates observed with SE signal and BSE signal. (a) SEM micrograph of the composite captured with the SE signal. (b) SEM micrograph of the composite captured with the BSE signal. Tilted angle SEM micrograph of the diamond/SiC composite. (f) SEM micrograph (SE signal) and corresponding EDS maps showing (d) Si and (e) C. Below, two EDS spectra are shown, signifying a Si-rich and a C-rich area of the SiC composite film. Figure S6: XRD diffraction pattern of diamond/silicon carbide composite film deposited on the flat WC-Co after the optimized pretreatment (15 min Murakami, 15 s Caro’s acid, and DND seeding) and TMS flow rate 80 sccm during CVD coating). Figure S7: Surface and cross-sectional morphology of the three coatings, namely, (a) microcrystalline diamond coating, (b) nanocrystalline diamond coating, and (c) composite interlayer + nanocrystalline diamond top layer. References [13,33,50,53,54,55,56,57] are cited in the Supplementary Materials.
