# Supplementary material for: Deposition of Diamond Coatings on Ultrathin Microdrills for PCB Board Drilling
Source: Materials (Basel). 2024 Nov 15;17(22):5593. doi: 10.3390/ma17225593 (PMC11595679; doi:10.3390/ma17225593)
Supplement: Supplementary file 1 [file materials-17-05593-s001.zip › materials-3312084-supplementary.pdf]

Deposition of Diamond Coatings on Ultrathin Microdrills for PCB Board Drilling

Shuangqing Zhou,[1] Stephan Handschuh-Wang,[2,*] Tao Wang,[1,3,*]

[1] Advanced Energy Storage Technology Center, Shenzhen Institutes of Advanced Technology, Chinese Academy of Sciences, Shenzhen 518055, China

[2] College of New Materials and New Energies, Shenzhen Technology University, Shenzhen 518118, China

[3] Advanced Materials Group Co., Ltd., Fusionopolis Link #06-07, Nexus One-North, Singapore 138543, Singapore

Supporting information

The substrate material of the microdrills is WC-Co. In this composite alloy, Co is an indispensable binder. The metal carbide in the composite provides high stiffness and hardness (wear resistance), while the cobalt binder endows the composite with ductility and toughness. This combination of properties is the reason for the wide industrial application of WC-Co.[1, 2] To improve the wear resistance, a diamond coating can be applied with the aid of CVD techniques. However, cobalt will catalyze the formation of graphite in the process of diamond deposition, reducing the purity and quality of diamond, and affecting the adhesion of the coating. Further, the Co moves with the growing diamond coating, deteriorating the overall growth, not only the initial growth. The adhesion is affected due to the initial formation of a graphitic phase catalyzed by the Co, other metals, such as Fe and Ni also catalyze the graphitic layer formation. Therefore, the Co binder is removed (depleted) superficially from the WC-Co for diamond deposition by treatment with Murakami solution, which leads to roughening of the WC-Co by dissolution of tungsten carbide grains, followed by acid treatment to remove the Co binder phase by dissolution and forming $CoSO_4$.[3] Though this method has been proposed in a patent in 1992,[4] a general treatment procedure has not been proposed conclusively, as the treatment times (and concentrations of the base and acid) have to be varied according to the alloy composition and grain size, and for filigree substrates shorter treatment times are necessary to avoid embrittlement of the tool. For instance, microdrills are known to become brittle after extended Co leaching, fracture (perhaps at the shaft shank) during drilling of PCB boards is therefore encountered rather than wearing down of deposited diamond coating (under the assumption that the diamond coating adheres to the tool). Therefore, a reasonable compromise between necessary Co-depletion (etching time) for diamond growth on the one hand and maintaining toughness by limiting the etching time have to be established.

At first, a flat WC-Co substrate with a Co content of approximately 6 at% was chosen. For this substrate, the Co depletion process was optimized. The surface morphology of the WC-Co substrate is shown in Figure S1a. The bright regions denote WC grains while the darker regions denote the binder Co. The as-received (polished) WC-Co substrate appears very smooth. Less binder is visible than WC, which agrees with the elemental composition determined by EDS, as

shown in Figure S1b. The composition determined by EDS is 79.4 at% W, 15.3 at% C, and 5.3 at% Co.

The change in the surface morphology and elemental composition of cemented carbide (WC-Co with 6 at% Co) after etching with the Murakami solution is shown in Figure S2a-d. The SEM micrographs signify that the surface of the alloy is roughened by Murakami etching. The roughness appears to increase for samples etched for 5 min and 10 min. There is little visible difference in the samples for longer etching times, such as 15 and 20 min. To unravel the composition of the Murakami-etched samples, EDS spectra and the elemental composition were measured and are shown below the corresponding SEM image. The EDS spectra signify an increase in peak intensity of Co. To observe the change in composition of the WC-Co substrate, in Figure S2e, the composition of the composite was plotted versus the Murakami etching time. Figure S2e provides evidence of dissolution of WC grains as the content of W in the composite is reduced from 79.4 at% to ca. 52 at% after 15 min. This dissolution of WC with Murakami solution is in line with literature.[3] Similarly, the content of C dwindles from 15.3 at% to ca. 1.5 at% after 15 min of etching. Conversely, the content of Co increases from 5.3 at% to ca. 42 at% after 15 min etching. Notably, continued etching after 15 min does not significantly change the surface composition of the composite alloy. Therefore, a Murakami etching time of 15 min was chosen as the optimized etching parameter for flat WC-Co substrates in our study. We assume that longer etching may result in deterioration of diamond film adhesion and embrittlement due to generation of cavities by dissolution of excess WC in a buried layer of the alloy.

As stated above, the substrate became more uneven (rougher) after etching, because the WC particles were dissolved. This in turn exposes the cobalt binder between the WC particles and makes dissolution of cobalt in a subsequent step easier (and faster). The increased roughness also provides more low-energy nucleation sites for the nucleation of the diamond, increases the contact area between the diamond coating and the substrate, and facilitates mechanical locking, which increases adhesion of CVD diamond coatings.

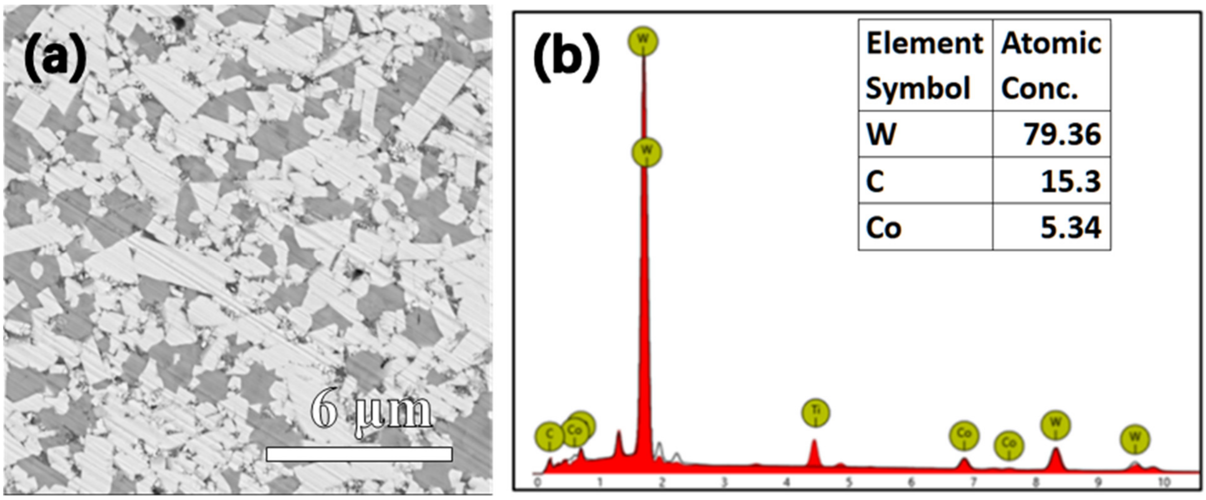

Figure S1 (a) SEM surface morphology of as-received WC-Co substrate. (b) EDS spectrum of as-received WC-Co substrate.

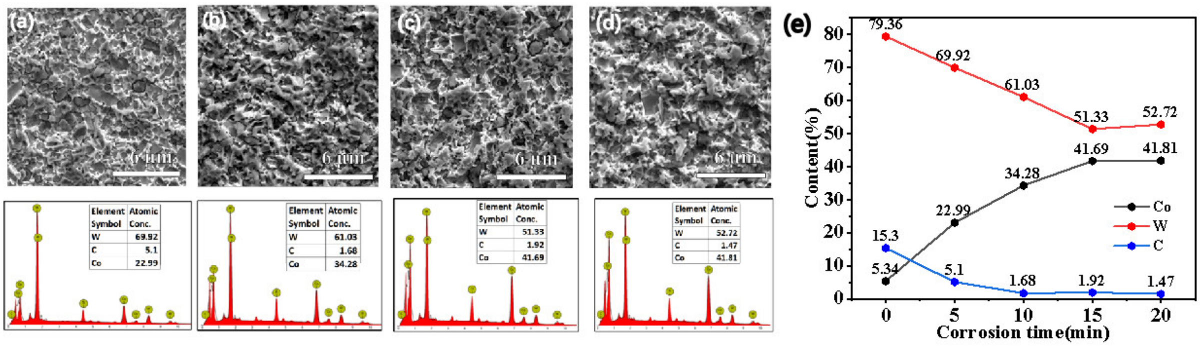

Figure S2 The surface morphology of the WC-Co substrate after etching for (a) 5 min, (b) 10 min, (c) 15 min, and (d) 20 min with Murakami solution. Below the SEM micrographs the corresponding EDS spectra are shown. (e) Composition of the etched WC-Co substrates determined by EDS.

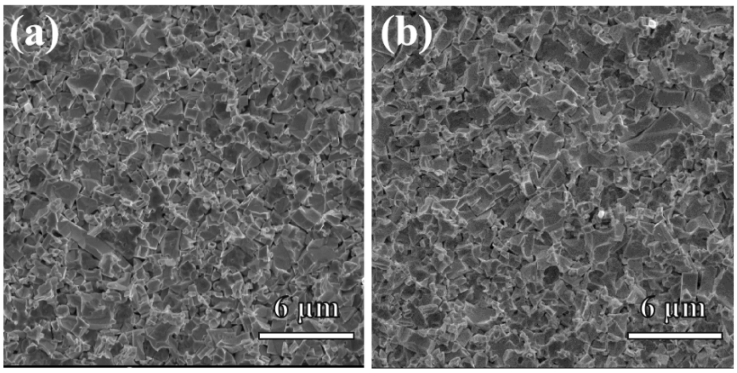

Figure S3 SEM morphology of a 0.125 mm WC-Co microdrill after Murakami etching for (a) 6 and (b) 9 min, followed by acid etching for 15 s.

Reason for using a SiC/diamond interlayer:

The large mismatch in thermal expansion coefficient is a challenge for the deposition of diamond on WC-Co substrates, as this mismatch leads to high thermal stresses. High thermal stresses lead to spallation of diamond coatings even though the adhesion strength might be high. To avoid stress concentration, interlayers have been used. The thermal expansion coefficient of silicon carbide is between that of cemented carbide and diamond. Adding silicon carbide to the diamond coating or using silicon carbide as the transition layer between the diamond coating and the substrate effectively lowers thermal stress caused by thermal expansion mismatch and improve the adhesion of the coating. The diamond/SiC composite films between the diamond coating and the substrate reduces thermal stresses arising from cooling down the microdrills from the deposition temperature and thus, improve adhesion of the diamond coating at temperatures close to room temperature.

Deposition of diamond/silicon carbide composite interlayer

Figure S4 shows diamond/SiC composite films grown at different tetramethylsilane (TMS) flow rates. When the flow rate of tetramethylsilane is between 20 and 60 sccm, discontinuous silicon carbide growth in the composite film is observed, as shown in Figure S4(a-c). At a flow rate of 80 sccm, silicon carbide grows continuously (Figure S4(d)). It can be seen from Figure S4d that SiC (darker) is distributed in blocks between diamond grains (brighter), with small particles protruding from the surface. The grown particles are relatively smooth and little granular particles are observed. At this flow rate, the ratio between diamond and SiC is close to 1:1, which is suitable for a composite interlayer. Further increasing the TMS flow rate to 100 sccm, results in preferential generation of SiC and thus, the SiC content is higher than the diamond content in Figure S4e. The diamond grains are located at the grain boundaries of the SiC. As the composition of this layer is depleted of diamond, it is not suitable as an interlayer. Based on this evaluation, the optimized deposition parameter for the diamond/SiC composite film is a TMS flow rate of 80 sccm during CVD deposition.

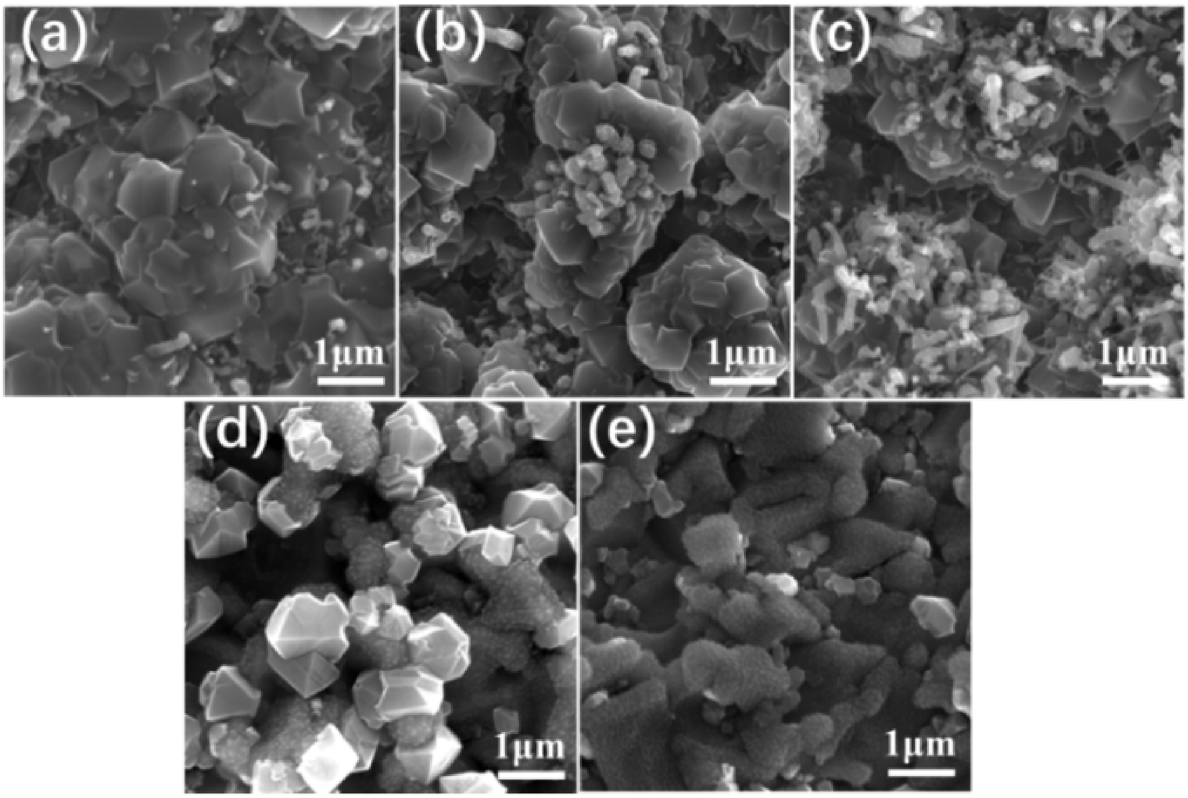

Figure S4 Surface morphology of diamond/silicon carbide composite films deposited on flat WC-Co sample after 1. etching for 15 min with Murakami and 15 s with Caro's acid and 2. seeding with milled DNDs. The TMS flow rates were (a) 20 sccm, (b) 40 sccm, (c) 60 sccm, (d) 80 sccm, (e) and 100 sccm.

Figure S5 shows the surface morphology of the diamond/SiC composite film made with a flow rate of 80 sccm TMS during CVD growth, captured with the secondary electron (SE) signal and back scattering electron (BSE) signal. The BSE image (Figure S5b) shows dark and bright areas, the dark areas denote diamond, as the relative molecular mass of C is lower compared to Si. The bright areas are SiC. Figure S5a, c, and f are secondary electron images and show the morphology of the composite coating. Further, EDS mapping of the area shown in Figure S4f was conducted, indicating the distribution of the elements Si (Figure S5d) and C (Figure S5e), respectively. The EDS mappings show that the grown crystallites are either rich in C or composed of a mixture of Si and C, which is corroborated with the EDS spectra of the C-rich and SiC-rich areas. The EDS spectra are shown in Figure S5f. From the SEM micrograph in Figure S5f, crystallites are visible. The content of the crystals matches the material distribution in Figure S5b.

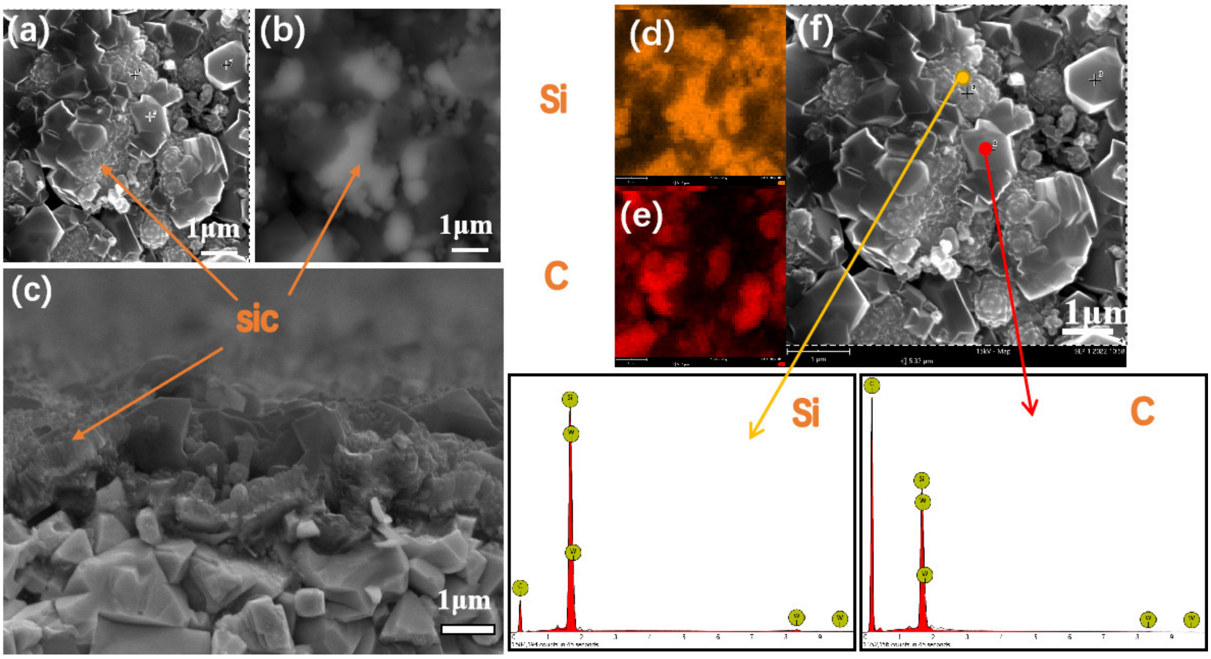

Figure S5 Surface morphology of diamond/SiC composite films made with 80 sccm TMS on flat WC-Co substrates observed with SE signal and BSE signal. (a) SEM micrograph of the composite captured with the SE signal. (b) SEM micrograph of the composite captured with the BSE signal. Tilted angle SEM micrograph of the diamond/SiC composite. (f) SEM micrograph (SE signal) and corresponding EDS maps showing (d) Si and (e) C. Below, two EDS spectra are shown, signifying a Si-rich and a C-rich area of the SiC composite film.

Figure S6 shows the XRD diffraction patterns of the diamond/SiC composite film deposited on WC-Co (80 sccm TMS) after the optimized etching procedure. XRD penetration depth is a few microns, while the composite coating is thinner (ca. 2.5 μm). Therefore, diffraction patterns from the underlying substrate are visible. These patterns are denoted with black squares in the diffraction pattern. The first three strong diffraction peaks related to the WC phase denote the lattice planes (001), (100), and (101).[5-7] In addition to the peaks of the WC alloy matrix, the diffraction peaks at 43.9° and 75.3° denote the lattice planes (111) and (220) of diamond, respectively.[6] The diffraction peaks at 59.72° and 71.4° denote the lattice planes (220) and (311) of SiC, respectively.[6, 8] In addition, there are diffraction peaks for the β-SiC (111) plane and diamond (220) plane corresponding to 35.3° and 75.2°, which overlap with the (100) and (200) plane diffraction peaks of the WC alloy, respectively. Therefore, the formation of diamond and SiC in the composite films is confirmed.

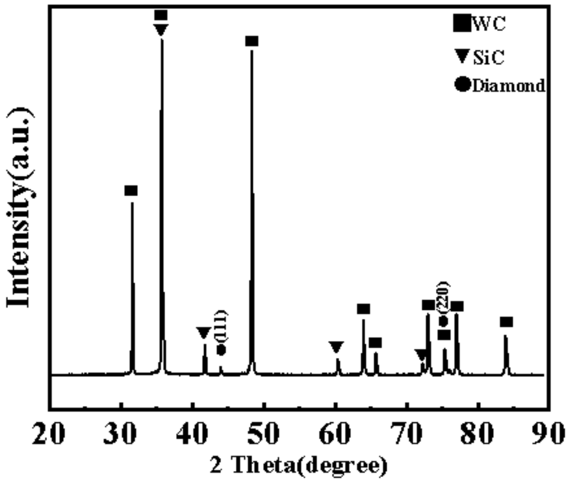

Figure S6 XRD diffraction pattern of diamond/silicon carbide composite film deposited on the flat WC-Co after the optimized pretreatment (15 min Murakami, 15 s Caro's acid, and DND seeding) and TMS flow rate 80 sccm during CVD coating).

Figure S7 shows the surface and cross-sectional morphology of the three coatings, namely microcrystalline, nanocrystalline, and composite coating (diamond/SiC interlayer and nanocrystalline diamond top layer), after optimized etching procedure. Figure 7a1 shows the surface morphology of a single-layer microcrystalline diamond coating with crystal sizes between 500 and 1000 nm. From the SEM micrograph it can be seen that the diamond grains have grain orientations of (100) and (111). Figure 7a2 shows the corresponding cross-sectional morphology, indicating good interfacial bonding. The thickness of the coating is between 2 and 2.5 µm. Similarly, Figure 7b1 shows the surface morphology of the nanocrystalline diamond coating, which is composed of large crystal clusters with a smooth surface and is cauliflower-like. The crystal size of the nanocrystalline coating is ≈ 20 nm. Corresponding to this coating, Figure 7b2 shows the cross-sectional morphology, signifying good interfacial bonding due to a lack of cavities. The coating features a thickness of 2.5 to 3 µm. Finally, Figure 7c1 shows the composite coating, comprising WC-Co substrate, microcrystalline diamond/SiC composite interlayer, and nanodiamond top layer. Figure 7c2 shows the corresponding cross-sectional morphology, signifying the presence of the bilayer system. The diamond/SiC composite interlayer is approx. 1 µm thick and fills the cavities of the WC-Co after etching, which is suggested to improve

interfacial adhesion of the final coating due to mechanical interlocking.[40] This filling of voids of the WC-Co can be seen in Figure 7c2 in the inset (EDS cross-sectional mapping). The final nanocrystalline coating has a thickness between 1 and 1.5 µm. Therefore, the overall thickness of all coatings is comparable. Good interfacial adhesion of these coatings is expected due to removal of the Co by etching (lack of detrimental effects of Co presence, i.e., avoiding graphitization), formation of carbides between the metal matrix and the coating, and the absence of cavities. The latter points to a relatively high seeding density of DNDs after the seeding step.

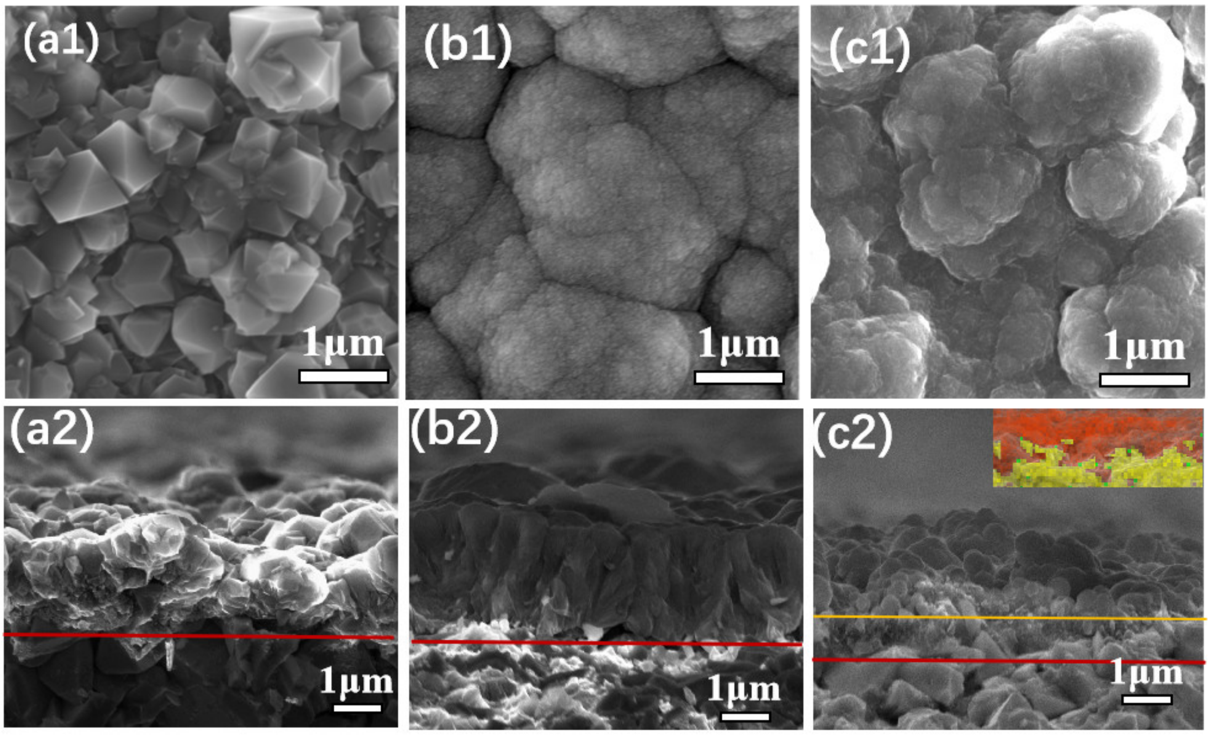

Figure S7 Surface and cross-sectional morphology of the three coatings, namely, (a) microcrystalline diamond coating, (b) nanocrystalline diamond coating, and (c) composite interlayer + nanocrystalline diamond top layer.

References:

(1) Boukantar, A.-R.; Djerdjare, B.; Guiberteau, F.; Ortiz, A. L. A critical comparison of the tribocorrosive performance in highly-alkaline wet medium of ultrafine-grained WC cemented carbides with Co, Co+Ni, or Co+Ni+Cr binders. *International Journal of Refractory Metals and Hard Materials* **2021**, *95*, 105452. DOI: https://doi.org/10.1016/j.ijrmhm.2020.105452.
(2) Yang, Y.; Zhang, C.; Wang, D.; Nie, L.; Wellmann, D.; Tian, Y. Additive manufacturing of WC-Co hardmetals: a review. *The International Journal of Advanced Manufacturing Technology* **2020**, *108* (5), 1653-1673. DOI: 10.1007/s00170-020-05389-5.

(3) Haubner, R.; Kubelka, S.; Lux, B.; Griesser, M.; Grasserbauer, M. Murakami and H2SO4/H2O2 Pretreatment of WC-Co Hard Metal Substrates to Increase the Adhesion of CVD Diamond Coatings. *J. Phys. IV France* **1995**, *05* (C5), C5-753-C755-760.
(4) Peters, M. G.; Cummings, R. H. Methods for coating adherent diamond films on cemented tungsten carbide substrates. EP0519587A1, 1992.
(5) Zhu, W.; Ignaszak, A.; Song, C.; Baker, R.; Hui, R.; Zhang, J.; Nan, F.; Botton, G.; Ye, S.; Campbell, S. Nanocrystalline tungsten carbide (WC) synthesis/characterization and its possible application as a PEM fuel cell catalyst support. *Electrochimica Acta* **2012**, *61*, 198-206. DOI: https://doi.org/10.1016/j.electacta.2011.12.005.
(6) Wang, T.; Xiang, L.; Shi, W.; Jiang, X. Deposition of diamond/β-SiC/cobalt silicide composite interlayers to improve adhesion of diamond coating on WC–Co substrates by DC-Plasma Assisted HFCVD. *Surface and Coatings Technology* **2011**, *205* (8), 3027-3034. DOI: https://doi.org/10.1016/j.surfcoat.2010.11.014.
(7) Wang, T.; Zhuang, H.; Jiang, X. One step deposition of highly adhesive diamond films on cemented carbide substrates via diamond/β-SiC composite interlayers. *Applied Surface Science* **2015**, *359*, 790-796. DOI: https://doi.org/10.1016/j.apsusc.2015.10.165.
(8) Cabral, G.; Gäbler, J.; Lindner, J.; Grácio, J.; Polini, R. A study of diamond film deposition on WC–Co inserts for graphite machining: Effectiveness of SiC interlayers prepared by HFCVD. *Diamond and Related Materials* **2008**, *17* (6), 1008-1014. DOI: https://doi.org/10.1016/j.diamond.2008.03.017.
